# Effects of *N*-acetylcysteine (NAC) supplementation in resuscitation fluids on renal microcirculatory oxygenation, inflammation, and function in a rat model of endotoxemia

**DOI:** 10.1186/s40635-016-0106-1

**Published:** 2016-09-26

**Authors:** Bulent Ergin, Philippe Guerci, Lara Zafrani, Frank Nocken, Asli Kandil, Ebru Gurel-Gurevin, Cihan Demirci-Tansel, Can Ince

**Affiliations:** 1 Department of Translational Physiology, Academic Medical Center, University of Amsterdam, Meibergdreef 9, 1105 AZ Amsterdam, The Netherlands; 2 University of Lorraine, Vandoeuvre-Lès-Nancy, France; 3 Divisional Medical & Clinical Affairs Generics & Standard Solutions, Volume Therapy, Fresenius Kabi Deutschland GmbH, Berlin, Germany; 4 Department of Biology, Faculty of Science, University of Istanbul, Istanbul, Turkey; 5 Department of Intensive Care, Erasmus MC, University Medical Center, Rotterdam, The Netherlands

**Keywords:** Acute kidney injury, Sepsis, *N*-acetylcysteine, Kidney oxygenation, Inflammation

## Abstract

**Background:**

Modulation of inflammation and oxidative stress appears to limit sepsis-induced damage in experimental models. The kidney is one of the most sensitive organs to injury during septic shock. In this study, we evaluated the effect of *N*-acetylcysteine (NAC) administration in conjunction with fluid resuscitation on renal oxygenation and function. We hypothesized that reducing inflammation would improve the microcirculatory oxygenation in the kidney and limit the onset of acute kidney injury (AKI).

**Methods:**

Rats were randomized into five groups (*n* = 8 per group): (1) control group, (2) control + NAC, (3) endotoxemic shock with lipopolysaccharide (LPS) without fluids, (4) LPS + fluid resuscitation, and (5) LPS + fluid resuscitation + NAC (150 mg/kg/h). Fluid resuscitation was initiated at 120 min and maintained at fixed volume for 2 h with hydroxyethyl starch (HES 130/0.4) dissolved in acetate-balanced Ringer’s solution (Volulyte) with or without supplementation with NAC (150 mg/kg/h). Oxygen tension in the renal cortex (CμPO_2_), outer medulla (MμPO_2_), and renal vein was measured using phosphorimetry. Biomarkers of renal injury, inflammation, and oxidative stress were assessed in kidney tissues.

**Results:**

Fluid resuscitation significantly improved the systemic and renal macrohemodynamic parameters after LPS. However, the addition of NAC further improved cortical renal oxygenation, oxygen delivery, and oxygen consumption (*p* < 0.05). NAC supplementation dampened the accumulation of NGAL or L-FABP, hyaluronic acid, and nitric oxide in kidney tissue (*p* < 0.01).

**Conclusion:**

The addition of NAC to fluid resuscitation may improve renal oxygenation and attenuate microvascular dysfunction and AKI. Decreases in renal NO and hyaluronic acid levels may be involved in this beneficial effect. A therapeutic strategy combining initial fluid resuscitation with antioxidant therapies may prevent sepsis-induced AKI.

## Background

Inflammation is a key process in the pathophysiology of septic shock [1]. The whole activation of leukocytes, the cascade of inflammation, the associated cytokine storm, and endothelial cell dysfunction collaborate to alter the microcirculation [2, 3]. Subsequently, tissue hypoxia and dysoxia due to heterogeneity of the microcirculation will occur [4]. Fluid resuscitation may not only help resolve some of these issues but also lead to the activation of oxidative pathways in itself, resulting in a heterogeneous distribution of blood flow and tissue oxygenation, especially in the renal cortex [5, 6]. Because reactive oxygen species (ROS) participate in the pathophysiology of endotoxemic shock, it has been suggested that moderating the oxidative stress and inflammatory reaction would translate into improving the microcirculation and oxygenation of the tissues. Previous studies have demonstrated an interest in the use of antioxidants for preventing sepsis-induced damage in these organs [7–9]. Indeed, the experimental literature is full of studies of drugs that target and are effective at dampening inflammation and oxidative stress. Some trials have reported several anti-inflammatory or antioxidant drugs with discordant effects on major outcomes [10, 11]. In fact, antioxidant therapies for specific organs might be of interest. Acute kidney injury (AKI) and acute lung injury are particularly common complications of sepsis, and the development of either increases mortality probably because these organs are more sensitive to inflammation and oxidative stress insults. Thus, the kidney could benefit from antioxidant drugs.

A common approach for the inhibition of oxidant-mediated injury is the use of glutathione-modulating agents such as sulfhydryl or thiol compounds. Among all the drugs used to interact with this pathway, *N*-acetylcysteine (NAC) is the most studied for its lung and renal protective effects [12–16]. NAC is a thiol compound with antioxidant and vasodilatory properties [12]. NAC is regarded as an important antioxidant as it is a source of sulfhydryl and glutathione groups in cells and, due to its interaction with ROS, is a scavenger of free radicals. In septic patients, the endogenous antioxidant glutathione is depleted [17]. Decreased levels of glutathione may lead to decreased protection of cell membranes against oxygen radicals. NAC serves as a precursor of glutathione and can replenish the intracellular glutathione stores [12]. Moreover, NAC targets kidney microcirculatory blood flow [18, 19]. NAC has also been widely studied for its nephroprotective effects in various settings.

Hypoxia and inflammation have an interdependent relationship. Several molecular pathways of cross-talk between hypoxia and inflammation in the kidney have been identified [20–22]. From a physiological perspective, although hypoxia may lead to inflammation and vice versa, it is unclear whether correcting or modulating either of these states would translate into better tissue oxygenation and improved outcomes. NAC would be interesting for testing whether the modulation of inflammation could correct tissue hypoxia during sepsis.

To date, no study has focused on tissue oxygenation in specific organs such as the kidney but rather demonstrated that either pretreatment or post-treatment with NAC decreased the markers of organ injury. In this study, we assessed kidney tissue oxygenation in an endotoxemic shock model resuscitated with balanced hydroxyethyl starch—Ringer’s acetate either with or without supplementation with NAC. We sought to promote blood flow and oxygenation to the organs by the means of reducing inflammation and oxidative stress.

## Methods

### Animals

All experiments in this study were approved by the institutional Animal Experimentation Committee of the Academic Medical Center of the University of Amsterdam (DFL102538). The care and handling of the animals were in accordance with the guidelines for Institutional Animal Care and Use Committees. The study was conducted in accordance with the Declaration of Helsinki. Experiments were performed on albino Wistar rats (Harlan Netherlands BV, Horst, The Netherlands) with a mean ± SD body weight of 325 ± 6 g.

### Surgical preparation

All animals were anesthetized with an intraperitoneal injection of a mixture of 90 mg/kg ketamine (Nimatek®, Eurovet, Bladel, The Netherlands), 0.5 mg/kg dexmedetomidine (Dexdomitor, Pfizer Animal Health BV, Capelle aan den IJssel, The Netherlands), and 0.05 mg/kg atropine-sulfate (Centrafarm Pharmaceuticals BV, Etten-Leur, The Netherlands). After a tracheotomy was performed, the animals were mechanically ventilated with a fraction of inspired oxygen (FiO_2_) of 0.4. Body temperature was maintained at 37 ± 0.5 °C during the entire experiment by an external thermal heating pad. Ventilator settings were adjusted to maintain an arterial partial pressure of carbon dioxide (PaCO_2_) between 35 and 40 mmHg. For drug and fluid administration as well as hemodynamic monitoring, vessels were cannulated with polyethylene catheters with an outer diameter of 0.9 mm (Braun, Melsungen, Germany). A catheter in the right carotid artery was connected to a pressure transducer to monitor the mean arterial blood pressure (MAP) and heart rate. The right jugular vein was cannulated for continuous infusion of Ringer’s lactate (Baxter, Utrecht, The Netherlands) at a rate of 15 ml/kg/h and for the maintenance of anesthesia. The right femoral artery was cannulated for drawing blood samples; the right femoral vein, for drug administration. The left kidney was exposed, decapsulated, and immobilized in a Lucite kidney cup (K. Effenberger, Pfaffingen, Germany) via an ~4-cm incision in the left flank in each animal. Renal vessels were carefully separated to preserve the nerves and adrenal gland. A perivascular ultrasonic transient time flow probe was placed around the left renal artery (type 0.7 RB Transonic Systems Inc., Ithaca, NY, USA) and connected to a flow meter (T206, Transonic Systems Inc., Ithaca, NY, USA) to continuously measure renal blood flow (RBF). The left ureter was isolated, ligated, and cannulated with a polyethylene catheter for urine collection. After the surgical preparation, one optical fiber was placed 1 mm above the decapsulated kidney, and another optical fiber was placed 1 mm above the renal vein to measure renal microvascular and venous oxygenation using phosphorimetry. A small piece of aluminum foil was placed on the dorsal side of the renal vein to prevent the underlying tissues from contributing to the phosphorescence signal in the venous PO_2_ measurements. The surgical field was covered with a humidified gauze compress throughout the entire experiment to prevent drying of the exposed tissues.

### Experimental protocol

**Fig. 1:**
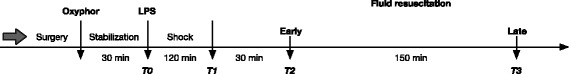
Timeline of the experimental protocol

After a 30-min stabilization, the rats were randomized into the five following groups at baseline: (1) control group, (2) control + NAC, (3) endotoxemic shock with lipopolysaccharide (LPS) without fluid resuscitation, (4) LPS + fluid resuscitation, and (5) LPS + fluid resuscitation + NAC (150 mg/kg/h). The groups received either an intravenous bolus of 5 mg/kg LPS (LPS group; *Escherichia coli* 0127:B8, Sigma, Paris, France; three groups of eight rats each) or vehicle (control group, two groups of eight rats each). Animals were observed or kept in shock for over 120 min. Fluid resuscitation (15 ml/kg/h) was then started and maintained for 180 min in the LPS groups with 6 % hydroxyethyl starch (HES130/0.4) dissolved in Ringer’s acetate (HES-RA; Volulyte® 6 %, Fresenius Kabi Deutschland GmbH, Germany) as a balanced colloid solution. NAC was administered to the appropriate groups at a rate of 150 mg/kg/h as previously reported [15]. An LPS group was not resuscitated to serve as a shock control. Time points for the measurements were baseline (T_0_), during shock 120 min after administration of LPS (T_1_), 30 min after initiating fluid resuscitation (early reperfusion phase) (T_2_), and 120 min after starting fluid resuscitation (late reperfusion phase) (T_3_), which was the final endpoint of the experiment (Fig. 1).

### Blood gas measurements and biochemistry

Arterial blood samples of 0.5 ml were collected from the femoral artery at T_0_, T_1_, T_2_, and T_3_. The blood samples were replaced by the same volume of balanced colloid solution. The samples were used to determine blood gas parameters (Radiometer ABL 505 Blood Gas Analyzer, Copenhagen, Denmark). The hematocrit and the levels of potassium, bicarbonate, and the anion gap were recorded by the analyzer.

### Measurement of renal microvascular oxygenation and venous PO_2_

The renal microvascular partial pressure of oxygen (μPO_2_) and renal venous PO_2_ (rvPO_2_) were measured by oxygen-dependent quenching of phosphorescence lifetimes of the phosphorescent dye Oxyphor G2 (Oxygen Enterprises Ltd., Philadelphia, PA, USA) as described previously [23, 24]. A total of 6 mg/kg IV over 5 min was administered followed by 30 min of stabilization before recording baseline measurements.

### Calculation of derivatives of oxygenation parameters and renal vascular resistance

Renal oxygen delivery was calculated using the following formula: DO_2ren_ (ml/min) = RBF × arterial oxygen content (1.31 × hemoglobin × SaO_2_) + (0.003 × PaO_2_), where SaO_2_ is the arterial oxygen saturation and PaO_2_ is the arterial partial pressure of oxygen. Renal oxygen consumption was calculated using the following formula: VO_2ren_ (ml/min/g) = RBF × (CaO_2_ − CvO_2_), where the renal venous oxygen content (CvO_2_) was calculated as (1.31 × hemoglobin × SrvO_2_) + (0.003 × rvPO_2_). The SrvO_2ren_ was calculated using the Hill equation with P_50_ = 37 Torr (4.9 kPa) and the Hill coefficient = 2.7. An estimation of the renal vascular resistance (RVR) was defined as RVR (dynes sec cm^−5^) = (MAP/RBF) × 100.

### Assessment of kidney function

Creatinine clearance (Cl_crea_ [ml/min]) was assessed as an index of the glomerular filtration rate. Clearance was calculated using the following formula: Cl_crea_ = (*U*_crea_ × *V*)/*P*_crea_, where *U*_crea_ is the concentration of creatinine in urine, *V* is the urine volume per unit time, and *P*_crea_ is the concentration of creatinine in the plasma. The renal energy efficiency for sodium transport (VO_2_/TNa) was assessed using a ratio calculated from the total amount of VO_2_ over the total amount of sodium reabsorbed (TNa, mmol/min) according to the following formula: (Cl_crea_ × PNa) − UNa × *V*.

### NO metabolism

The index of total nitric oxide (NO) production is the sum of both nitrite and nitrate accumulated in tissue samples. To determine this index, a saturated solution of vanadium (III) chloride (VCl_3_) in 1 mol/l HCl was used as a reducing agent. At a temperature of 90 °C, the VCl_3_ reagent quantitatively converts nitrite, nitrate, and S-nitroso compounds to NO in a glass reaction vessel. NO was then flushed out of the reaction vessel by the flow of helium gas and was then measured using a Sievers NO analyzer (General Electric Company, GE Water & Process Technologies Analytical Instruments) to detect chemiluminescence as the amount of light from the ozone-NO reaction in the measurement chamber of the analyzer. NO levels were determined in homogenized frozen kidney tissues. A ratio of tissue NO to tissue protein content was used for standardization of NO release per gram of protein.

### Glycocalyx component assessments

Hyaluronan is the main component of the endothelial glycocalyx, and alterations in its concentration are attributed to glycocalyx volume loss. Inhibition of tumor necrosis factor-alpha protects against endotoxin-induced endothelial glycocalyx perturbation. Plasma hyaluronan concentrations were determined using a Corgenix hyaluronic acid test kit (Corgenix Inc., Westminster, CO, USA) based on an enzyme-linked hyaluronic acid-binding protein assay.

### Measurement of oxidative stress and inflammatory cytokines

All kidneys were homogenized in cold 5 mM sodium phosphate buffer. The homogenates were centrifuged at 12,000*g* for 15 min at 4 °C, and the supernatants were used to determine TNF-α, IL-6, hyaluronic acid, malondialdehyde (MDA), and protein carbonyl levels. The levels of these markers were expressed as per gram of protein (Bradford assay). To determine the oxidative stress and inflammatory cytokines levels, enzyme-linked immunosorbent assay (ELISA) kits were used. Tumor necrosis factor-α (TNF-α) (DY510, R&D system, Inc. Minneapolis, USA), interleukin-6 (IL-6) (DY506, R&D system, Inc. Minneapolis, USA), tissue MDA, and protein carbonyl were determined in homogenized tissue samples.

### Immunohistochemical analysis

Kidney tissues were fixed in 4 % formalin and embedded in paraffin. After preparation, kidney sections were incubated with a neutrophil gelatinase-associated lipocalin (NGAL) antibody (NGAL antibody 41105, Abcam, Cambridge, UK) and a polyclonal antibody to rat liver-type fatty acid protein (L-FABP) (HP8010, Hycult Biotect, Uden Holland). Antibodies were diluted in a large volume of UltrAb Diluent (TA-125-UD, Thermo Fisher Scientific, Breda, Holland). The slides were counterstained with Mayer’s hematoxylin (LabVision TA-125-MH Thermo Fisher Scientific, Breda, Holland) and mounted in a vision mount (LabVision, TA-060-UG, Thermo Fisher Scientific, Breda, Holland) after washing in distilled water. Both the intensity and the distribution of L-FABP and NGAL staining were scored. For each sample, a histological score (HSCORE) value was derived by summing the percentages of cells that were stained at each intensity multiplied by the weighted intensity of the staining: HSCORE = S Pi (*i* + 1), where *i* is the intensity score and Pi is the corresponding percentage of the cells.

### Statistical analyses

The results are expressed as the mean ± SD. Statistical significance was calculated by one-way and two-way analysis of variance (ANOVA) followed by either Tukey’s or Bonferroni’s multiple comparison tests using GraphPad Prism (GraphPad Prism, Version 5, Software Program, San Diego, CA, USA). *p* < 0.05 was considered statistically significant.

## Results

### Systemic and renal hemodynamic parameters

The evolution of systemic and renal hemodynamics is presented in Table 1. Infusion of LPS induced an early drop in the MAP (76.8 ± 9.3 mmHg versus 45.8 ± 7.9 mmHg, *p* < 0.001) and RBF (4.5 ± 1.5 ml/min versus 0.7 ± 0.6 ml/min, *p* < 0.001) in the control group versus the LPS group, respectively, at T_3_. Fluid resuscitation with HES-RA both with and without NAC significantly improved RBF compared to the LPS alone group (*p* < 0.05). Both HES-RA and HES-RA + NAC significantly decreased the RVR compared to LPS alone (*p* < 0.001). After LPS, the addition of NAC to the fluid did not result in improved hemodynamic parameters compared to fluid resuscitation alone. The infusion of NAC led to a decrease in the MAP in the absence of LPS (57.3 ± 5.6 mmHg versus 76.8 ± 9.3 mmHg in the control group, *p* < 0.001).

**Table 1:** Evolution of systemic and renal hemodynamics parameters during the experiment

	T_0_ (baseline)	T_1_ (shock)	T_2_ (30 min)	T_3_ (120 min)
MAP (mmHg)				
Time control	102 ± 9.0	86.3 ± 13.4	80.1 ± 8.8	76.8 ± 9.3
Control + NAC	87.7 ± 7.6	75.3 ± 10.5	70.5 ± 8.0	57.3 ± 5.6***
LPS	101 ± 11.1	56.8 ± 10.5***	53.7 ± 9.4***	45.8 ± 7.9***
LPS + HES-RA	92.1 ± 8.7	55.6 ± 9.5***	*65.5 ± 4.9* * ^**, +**^	51.6 ± 3.7***
LPS + HES-RA + NAC	98.8 ± 8.7	49.8 ± 8.5***	64.1 ± 4.7**	52.6 ± 6.1***
RBF (ml/min)				
Time control	5.4 ± 0.6	4.8 ± 0.8	5.3 ± 0.9	4.5 ± 1.5
Control + NAC	5.7 ± 0.7	4.3 ± 0.9	4.8 ± 1.5	4 ± 1.1
LPS	5.7 ± 1.4	1.3 ± 0.7***	1.4 ± 0.5***	0.7 ± 0.6***
LPS + HES-RA	6.6 ± 0.3	1.9 ± 1.5***	*5.6 ± 1.8* ^+++^	*5.0 ± 0.4* ^+++^
LPS + HES-RA + NAC	5.5 ± 0.4	1 ± 0.4***	3.5 ± 0.9*^, ++^	*4.8 ± 2.2* ^+++^
RVR )dyn.s.sec^−5^)				
Time control	1910.2 ± 260.5	1793.7 ± 227.4	1524.3 ± 188.2	1861.3 ± 751.2
Control + NAC	1548.6 ± 249.2	1782.6 ± 368.6	1606.7 ± 583.2	1521.9 ± 511.1
LPS	1774.8 ± 302.4	5911.5 ± 3333.4***	4182.9 ± 829.7*	*10,372 ± 5182.2* ***
LPS + HES-RA	1410 ± 152.2	*3293.2 ± 1978.9* ^+^	*1130.2 ± 348.1* ^++^	*1004.3 ± 48.3* ^+++^
LPS + HES-RA + NAC	1787.2 ± 236	5795.8 ± 2517.4***	*1909.8 ± 375.7*	*1199.1 ± 318.3* ^+++^

*LPS* lipopolysaccharide, *HES-RA* hydroxyethyl starch-ringer acetate, *NAC N*-acetylcysteine

Values are presented as mean ± SD *p < 0.05, **p < 0.01, and ***p < 0.001 control versus other groups; ^+^ *p* < 0.05, ^++^ *p* < 0.01, and ^+++^ *p* < 0.001 LPS versus other groups

### Renal microvascular oxygenation

**Fig. 2:**
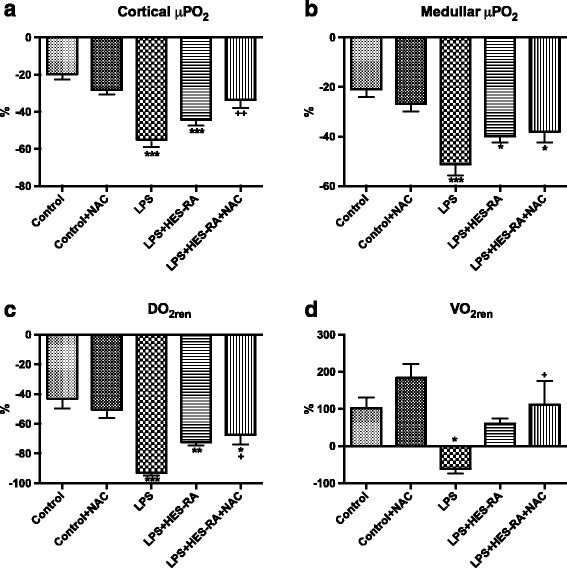
Percentage change of renal microvascular oxygen tension, oxygen delivery, and consumption from baseline to T_3_. In the renal cortex (CμpO_2_) (**a**), in the medulla (MμpO_2_) (**b**), renal oxygen delivery (DO_2ren_) (**c**), and renal oxygen consumption (VO_2ren_) (**d**). *p < 0.05, **p < 0.01, *p < 0.001 versus control; ^+^ *p* < 0.05 LPS versus LPS group

The percentage variations in CμPO_2_, MμPO_2_, DO_2ren_, and VO_2ren_ between baseline (T_0_) and the end of the experiment (T_3_) are shown in Fig. 2. Compared to the control groups, LPS infusion induced a significant decrease in CμPO_2_ (40.6 ± 8.8 mmHg versus 68.2 ± 4.1 mmHg in the control group at T_3_, *p* < 0.001) and MμPO_2_ (32.2 ± 7.9 mmHg versus 51.6 ± 3.2 mmHg in the control group, *p* < 0.001). Fluid resuscitation with HES-RA alone did not improve either CμPO_2_ or MμPO_2_. HES-RA combined with NAC significantly improved CμPO_2_ during sepsis (*p* < 0.01). LPS induced a significant decrease in DO_2ren_ and VO_2ren_ (8.3 ± 6.1 ml O_2_/min in the LPS group versus 67.2 ± 23.2 ml O_2_/min in the control group at T_3_ and 7.8 ± 6.5 ml O_2_/min in the LPS group versus 32.9 ± 10 ml O_2_/min in the control group at T_3_, *p* < 0.05, respectively). Fluid resuscitation with or without NAC significantly improved VO_2ren_ compared to the LPS alone group (*p* < 0.05). Of note, the addition of NAC to the control group also increased VO_2ren_ compared to the control group alone (*p* < 0.05). The hematocrit values are reported in Table 3. A significant decrease in hematocrit occurred after fluid resuscitation in both groups compared to the control and LPS alone groups (*p* < 0.001). The magnitude of hemodilution in both groups was in the same range.

LPS induced a significant increase in ER% O_2_ at T_1_, T_2_, and T_3_ compared to the control group (*p* < 0.01, *p* < 0.01, and p < 0.001, respectively), and this value was only improved in LPS + HES-RA group at T_2_. In the control group receiving NAC, ER%O_2_ was also increased at T_3_ compared to control (*p* < 0.001).

### Kidney function and biomarkers of kidney injury

The evolution of TNa^+^, VO_2ren_/TNa^+^, Cl_Crea_, and EFNa^+^ at different time points is presented in Table 2. TNa^+^ levels were lower in the LPS, LPS + HES-RA, and LPS + HES + RA + NAC groups (*p* < 0.001) than the control group at T_1_, T_2_, and T_3_. Fluid resuscitation did not restore these values. The addition of NAC also decreased the TNa + in the control + NAC group compared to the control group (*p* < 0.01). VO_2ren_/TNa^+^ increased in LPS groups receiving HES-RA and HES + RA + NAC at T_3_ compared to the control group (*p* < 0.05 and *p* < 0.001, respectively) and the LPS alone group (*p* < 0.05 and *p* < 0.001, respectively). Compared to the control and LPS alone groups, EFNa^+^ values were increased in the LPS groups treated with HES-RA with and without NAC at T_2_ and T_3_. NAC administration in the control group tended to decrease TNa^+^ and EFNa^+^ without reaching a significant level. Fluid resuscitation improved urine output regardless of the addition of NAC compared to the LPS group (*p* < 0.001). No effect of NAC on urine output was noted in the control + NAC group compared to the control group, but a significant decrease in Cl_creat_ was observed (*p* < 0.001).

**Table 2:** Parameters of renal function and excretion at different time points of the experiment

	T0 (baseline)	T1 (shock)	T2 (30 min)	T3 (120 min)
TNa^+^ (mmol/min^−1^)				
Time control	15.3 ± 9.6	14.1 ± 7.0	18.8 ± 6.8	19.7 ± 4.7
Control + NAC	15.0 ± 6.4	13.2 ± 6.5	15.9 ± 6.5	9.5 ± 4.3**
LPS	12.3 ± 5.5	0.00 ± 0.00***	0.00 ± 0.00***	0.00 ± 0.00***
LPS + HES-RA	12.0 ± 8.3	0.00 ± 0.00***	5.0 ± 1.12***	2.2 ± 1.3***
LPS + HES-RA + NAC	11.9 ± 5.7	0.00 ± 0.00***	2.8 ± 2.8***	2.2 ± 1.8***
VO_2_/TNa^+^				
Time control	1.77 ± 1.6	2.19 ± 1.3	1.59 ± 0.6	1.79 ± 0.7
Control + NAC	1.36 ± 0.4	2.19 ± 0.9	1.95 ± 1.18	6.32 ± 3.9
LPS	2.5 ± 1.2	0.00 ± 0.00	0.00 ± 0.00	0.00 ± 0.00
LPS + HES-RA	2.24 ± 1.1	0.00 ± 0.00	6.06 ± 1.7	17.17 ± 7.1*^, +^
LPS + HES-RA + NAC	1.97 ± 1.3	0.00 ± 0.0^*^	23.36 ± 22.2	28.53 ± 28***^, +++^
Cl_Crea_ (ml/min)				
Time control	0.11 ± 0.6	0.10 ± 0.04	0.14 ± 0.04	0.14 ± 0.03
Control + NAC	0.11 ± 0.04	0.09 ± 0.04	0.11 ± 0.04	0.06 ± 0.03***
LPS	0.09 ± 0.04	0.00 ± 0.00***	0.00 ± 0.00***	0.00 ± 0.00***
LPS + HES-RA	0.09 ± 0.06	0.00 ± 0.00***	0.05 ± 0.01***^, +^	0.02 ± 0.01***
LPS + HES-RA + NAC	0.09 ± 0.04	0.00 ± 0.00***	0.04 ± 0.01***	0.02 ± 0.01***
EFNa^+^				
Time control	4.8 ± 4.7	13.2 ± 5.7	11.9 ± 4.9	10.4 ± 2.4
Control + NAC	2.3 ± 0.8	3.22 ± 1.7	2.9 ± 1.7	6.9 ± 6.6
LPS	3.7 ± 1.4	0.00 ± 0.00	0.00 ± 0.00	0.00 ± 0.00
LPS + HES-RA	3.5 ± 2.8	0.00 ± 0.00	35.3 ± 7.3**^, +++^	17.1 ± 8.1
LPS + HES-RA + NAC	4.4 ± 3.9	0.00 ± 0.00	45.8 ± 36.7***^, +++^	31.4 ± 28.4***^, +++^
Urine volume (ml)				
Time control	0.39 ± 0.14	0.53 ± 0.13	0.45 ± 0.13	0.19 ± 0.03
Control + NAC	0.25 ± 0.09	0.34 ± 0.17	0.2 ± 0.04	0.16 ± 0.06
LPS	0.31 ± 0.13	0.06 ± 0.09***	0.04 ± 0.05***	0.02 ± 0.05***
LPS + HES-RA	0.37 ± 0.3	0.12 ± 0.19***	*0.6 ± 0.23* ^+++^	*0.16 ± 0.08* ^+++^
LPS + HES-RA + NAC	0.33 ± 0.26	0.04 ± 0.09***	*0.65 ± 0.47* ^+++^	*0.2 ± 0.13* ^+++^

Values are presented as mean ± SD

*TNa+* tubular sodium reabsorption, *Cl*_*creat*_ clearance of creatinine, *LPS* lipopolysaccharide, *HES-RA* hydroxyethyl starch-ringer acetate, *NAC N*-acetylcysteine

*p < 0.05, **p < 0.01, and ***p < 0.001 versus control; ^+^ *p* < 0.05 and ^+++^ *p* < 0.001 versus LPS

**Fig. 3:**
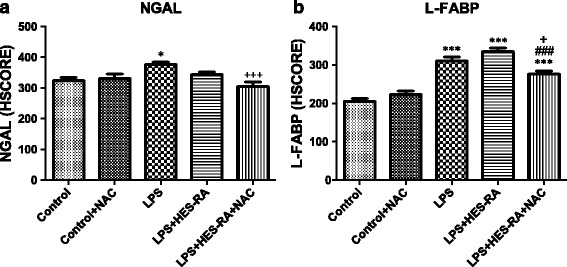
Immunostaining intensity (HSCORE) of NGAL (**a**) and L-FABP (**b**) in kidney cortex of all groups. *p < 0.05, ***p < 0.001 versus control group; +p < 0.05, +++p < 0.001 versus LPS group, ###p < 0.001 versus LPS + HES-RA group

Biomarkers of AKI (NGAL and L-FABP) were significantly increased in the kidney after LPS infusion (*p* < 0.05 and *p* < 0.001, respectively, versus the control group) (Fig. 3). The resuscitation fluid combined with NAC significantly decreased these biomarkers compared to the LPS alone group (*p* < 0.001 and <0.05, respectively). Moreover, L-FABP was lower in the group of septic rats resuscitated with HES-RA + NAC than with HES-RA alone (*p* < 0.001).

### Plasma electrolytes and acid-base status

Bicarbonate and plasma lactate levels, pH, base excess, and anion gap with K^+^ are shown in Table 3. LPS infusion significantly increased the plasma lactate level and anion gap, which could not be corrected by the administration of HES-RA either with or without NAC (*p* < 0.001) compared to the control group. Base excess, pH, and bicarbonate levels were similarly decreased after LPS infusion and were partially corrected by fluid resuscitation. NAC infusion alone in the control group resulted in a significant decrease in pH, base excess, and bicarbonate levels compared to the control group (*p* < 0.01). NAC administration worsened the acid-base status in the LPS resuscitated and control groups. The level of bicarbonate and base excess were significantly lower in rats resuscitated with fluid plus NAC than with fluid alone (*p* < 0.01).

**Table 3:** Time-course of acid-base status, lactate levels, and hematocrit resuscitated with and without the addition of NAC

	T0 (baseline)	T1 (shock)	T2 (30 min)	T3 (120 min)
pH				
Time control	7.37 ± 0.03	7.39 ± 0.06	7.42 ± 0.03	7.42 ± 0.04
Control + NAC	7.48 ± 0.12	7.42 ± 0.03	7.38 ± 0.02	7.32 ± 0.05*
LPS	7.36 ± 0.03	7.27 ± 0.06**	7.26 ± 0.07***	7.2 ± 0.07***
LPS + HES-RA	7.36 ± 0.03	7.26 ± 0.07***	7.31 ± 0.07*	7.27 ± 0.05***
LPS + HES-RA + NAC	7.37 ± 0.04	7.26 ± 0.09***	7.27 ± 0.08***	7.20 ± 0.03***
HCO_3_^−^ (mmol/L)				
Time control	20.6 ± 0.9	21 ± 0.6	21.2 ± 1.3	21.6 ± 1.3
Control + NAC	18.8 ± 0.6	18.4 ± 1.5*	19.3 ± 1.2	14.2 ± 3.8***
LPS	20.2 ± 0.7	15 ± 0.9***	15.3 ± 1.2***	12.8 ± 0.5***
LPS + HES-RA	21.6 ± 0.7	14.8 ± 1.5***	18.1 ± 0.9***^,^^++^	16.7 ± 1.6***, ^+++^
LPS + HES-RA + NAC	20.7 ± 0.8	14.7 ± 2.5***	16.6 ± 1.6***	14 ± 1.7***^, ##^
Base excess (mmol/L)				
Time control	−3.5 ± 1.2	−2.7 ± 1.6	−1.7 ± 1.3	−1.8 ± 1.5
Control + NAC	−3.5 ± 0.5	−4.4 ± 1.7	−4.5 ± 1.3	−8.9 ± 2.4***
LPS	−4 ± 1.3	−10.8 ± 1.9***	−10.5 ± 1.7***	−14.1 ± 1.3***
LPS + HES-RA	−2.9 ± 1.1	−11.3 ± 2.7***	−7 ± 2***^, ++^	−8.9 ± 1.9***^, +++^
LPS + HES-RA + NAC	−3.4 ± 1.5	−11.2 ± 4.1***	−9.1 ± 2.8***	−12.5 ± 1.7***^, ##^
Anion gap K^+^ (mmol/L)				
Time control	17.7 ± 0.8	18.0 ± 0.7	16.4 ± 0.5	16.8 ± 1.3
Control + NAC	18.9 ± 2.0	18.5 ± 2.0	17.0 ± 1.2	18.9 ± 4.8
LPS	18.9 ± 2.3	21.8 ± 1.1	21.6 ± 1.8	23.2 ± 1.6***
LPS + HES-RA	17.5 ± 1.3	22.7 ± 1.3	19.2 ± 1.0	20.9 ± 2.5***
LPS + HES-RA + NAC	18.4 ± 1.0	22.5 ± 2.5**	20.6 ± 2.3*	23.1 ± 1.9***
Lactate (mmol/L)				
Time control	2.32 ± 0.52	2.22 ± 0.34	2.08 ± 0.31	1.73 ± 0.27
Control + NAC	3.10 ± 0.64	2.92 ± 0.76	2.17 ± 0.36	2.10 ± 0.33
LPS	2.42 ± 0.32	*3.08 ± 0.28*	*3.08 ± 0.5*	4.32 ± 0.6***
LPS + HES-RA	1.92 ± 0.2	*3.32 ± 0.55*	*2.75 ± 0.48*	5.08 ± 1.83***
LPS + HES-RA + NAC	2.70 ± 0.21	3.83 ± 1.36**	*3.30 ± 1.17* ^*^	4.98 ± 1.21***
Hct (%)				
Time control	49.6 ± 1.3	41.6 ± 1.9	39 ± 2.9	33.3 ± 3
Control + NAC	48.8 ± 2.6	42.8 ± 4.4	41.1 ± 4.2	33.8 ± 2.6
LPS	49.3 ± 4.0	39.6 ± 3.0	35.1 ± 2.9	34.0 ± 3.5
LPS + HES-RA	49.3 ± 1.7	*43.3 ± 2.5*	30.1 ± 4.5***^, +^	17.5 ± 3***^, +++^
LPS + HES-RA + NAC	48.6 ± 0.8	*44.1 ± 3.2* ^+^	33.0 ± 2.6**	18.0 ± 2.0***^, +++^

*LPS* lipopolysaccharide, *HES-RA* hydroxyethyl starch-ringer acetate, *NAC N*-acetylcysteine

Values are presented as mean ± SD, *p < 0.05, **p < 0.01, ***p < 0.001 versus control; ^++^ *p* < 0.05, ^+++^ *p* < 0.001 versus LPS; ^##^ *p* < 0.01 versus LPS + HES-RA

### Oxidative stress and inflammatory cytokines

**Fig. 4:**
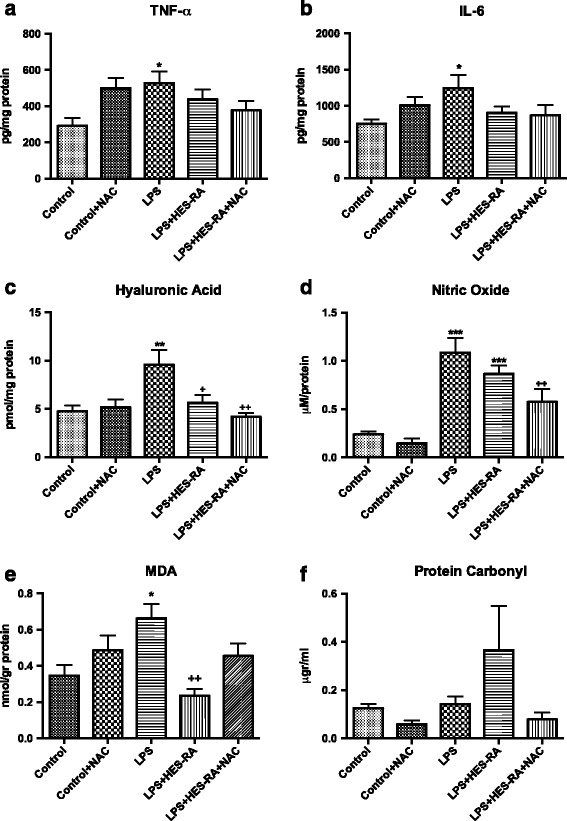
Levels of biomarkers of oxidative stress and pro-inflammatory cytokines in renal tissue. Renal tissue TNF-α (**a**), IL-6 (**b**), hyaluronic acid (**c**), nitric oxide (**d**), MDA (**e**), and protein carbonyl (**f**). *p < 0.05, **p < 0.01, ***p < 0.001 versus control; +*p* < 0.05, ++*p* < 0.01 versus LPS group

The levels of biomarkers of oxidative stress, pro-inflammatory cytokines, and products of glycocalyx degradation are represented in Fig. 4. The levels of TNF-α (**3A**) and IL-6 (**3B**) in kidney homogenates from the LPS group were significantly increased compared to the control group (528.1 ± 143.9 pg/mg protein versus 291.8 ± 99.1 pg/mg protein, *p* < 0.05; and 1246 ± 441 pg/mg protein versus 753.8 ± 122 pg/mg protein, *p* < 0.05, respectively). The same results were observed regarding hyaluronic acid (HA) (**3C**), nitric oxide (**3D**), and MDA (**3E**) after LPS infusion (*p* < 0.05). The addition of NAC to HES-RA during fluid resuscitation resulted in a significant lower level of HA and nitric oxide compared to the LPS group (*p* < 0.01). Infusion of HES-RA alone decreased the levels of MDA compared to LPS alone (*p* < 0.01) (**3E**). Protein carbonyl levels were not altered (**3E**).

## Discussion

In the present study, we found that fluid supplemented with NAC improved cortical renal oxygenation, oxygen delivery, and oxygen consumption compared to the LPS group. Fluid resuscitation alone was partially effective in correcting kidney hypoxia but did not reach a significant level compared to the LPS group. The addition of NAC to the resuscitation fluid did not further improve systemic or renal hemodynamics compared to HES-RA alone. It has been suggested that a specific effect of NAC on microvascular oxygenation exists independent of renal macrovascular perfusion. In an experimental study, Heyman et al. showed that NAC induced vasodilation in a pre-constricted renal microvasculature rat model [18]. The vasculature may be similarly constricted after LPS infusion leading to microcirculation heterogeneity [25]. Although creatinine levels did not differ between septic rats receiving fluids either with or without NAC, the fluid resuscitation combined with NAC decreased the levels of renal NO, hyaluronic acid, and early markers of acute kidney injury such as NGAL or L-FABP. Previous studies demonstrated a significant decrease in inflammatory biomarkers in specific organs such as the lungs and kidneys in models of sepsis [14–16, 26–29]. However, none of these studies reported the beneficial effects of NAC on tissue oxygenation during sepsis by decreasing oxidative stress and inflammation.

Several studies using microcirculatory techniques have now questioned the significance of arterial RBF and have focused on the renal microcirculation as the hemodynamic culprit in the pathophysiology of septic AKI [6, 25, 30]. Microcirculatory dysfunction may contribute to renal hypoxia even in the absence of overt renal hypoperfusion. The microcirculation of the renal cortex has been shown to be severely injured in animal models of sepsis. After LPS infusion in rats, Legrand et al. showed that fluid resuscitation could not fully restore renal microcirculatory dysfunction [6]. In dogs, endotoxemia was found to be associated with renal hypoperfusion and hypoxia in the renal cortex but was concomitant with increased renal venous PO_2_, supporting the concept that convective shunting of oxygen may contribute to the development of tissue hypoxia [31]. In our study, the LPS-induced renal microvascular heterogeneity and hypoxia appeared to be corrected with the NAC-supplemented fluid.

We also observed negative effects of NAC infusion. Delayed hypotension occurred in the control + NAC group, highlighting the vasodilatory effects of this compound as previously described [12, 32]. The mechanism involved in this lowering effect might be mediated by the interaction of sulfhydryl groups with enzymes such as the guanylate cyclase, which is the primary receptor for NO [32]. This drop in the MAP may contribute to tissue hypoperfusion and hypoxia as well as the lower pH observed in the control + NAC group compared to the control group. However, no significant change was observed in renal blood flow measured in the renal artery. Conflicting data exist between experimental and human clinical studies regarding the effects of NAC on regional blood flow and cardiac output [19, 33–37]. First, NAC administration was shown to improve survival in experimental models of peritonitis-induced sepsis [38, 39]. In mongrel dogs subjected to endotoxemia, Zhang et al. demonstrated the myocardial protective effects of NAC pretreatment (150 mg/kg) with enhanced oxygen delivery but lower systemic and pulmonary pressures [37]. In contrast, in a study involving 20 patients with septic shock, NAC infusion (150 mg/kg for 15 min followed by continuous infusion) that was initiated within 24 h after the onset of septic shock resulted in a decrease in left ventricular stroke work—revealing myocardial depression—without a significant impact on MAP at 48 h after treatment initiation [33]. In a similar population, Rank et al. demonstrated the exact opposite effect with an improvement in liver blood flow, oxygen delivery, and oxygen consumption related to an increase in cardiac index [34]. However, the infusion of NAC lasted less than 2 h in this latter study. Agustí et al. reported an increase in the cardiac index associated with vasodilatation but without improvements in splanchnic microcirculation after NAC infusion in patients presenting with septic shock and multiple organ failure [35]. Clinical studies yielded controversial results with the use of NAC in sepsis [11]. NAC treatment during the first hours of sepsis or septic shock may decrease peroxidative stress [40], improve hepatic function [34], and enhance tissue oxygenation and cardiac function [41], whereas delayed administration adversely affected the outcomes of critically ill patients with multiple organ failure [33, 42].

Most published studies have examined the effects of NAC when given as a pretreatment, e.g., before the insult. Here, we evaluated the ability of NAC when administered during the resuscitation process to correct tissue hypoxia and inflammation already present. It seems that the beneficial effects on tissue oxygenation, if any, are not similar if NAC is administered before or after the insult. The effects also depend on the time elapsed since the insult. We showed that NAC-supplemented fluid did not provide additional benefits on the acid status and renal function compared to fluid alone. Only kidney oxygenation was significantly higher in the group receiving fluid supplemented with NAC compared to the LPS alone group, whereas fluid alone did not reach a level of significance. Pretreatment with NAC appeared to be more efficient than post-injury treatment in protecting tissues against oxidative stress and inflammation in models of sepsis and ischemia/reperfusion injury. Due to the mechanisms of action of NAC, it is conceivable that it would be easier to prevent certain pathways from being activated rather than to modulate already highly activated signals with redundant or alternative pathways.

The effects of NAC on renal oxygenation could be mediated by different mechanisms. First, tissue NO levels were significantly increased after LPS infusion. By decreasing NO levels in the cortex, NAC combined with fluid administration may improve microvascular dysfunction, microvascular delivery of oxygen, and cortical oxygenation. Similarly, our group previously demonstrated that the prostaglandin analog iloprost restored kidney function in a rat model of endotoxemia and prevented the occurrence of hypoxic regions [43]. The improvement of renal microvascular oxygenation was mediated in part by the inhibition of inducible nitric oxide synthase expression in the kidney. Another major player involved in endothelial dysfunction in sepsis-induced AKI is the widespread damage to the endothelial glycocalyx, which may contribute to microvascular dysfunction via impaired flow-dependent vasodilatation. Hyaluronic acid levels reflect the disruption of the glycocalyx [44]. In the present study, hyaluronic acid levels were significantly increased after LPS infusion. Fluid resuscitation either with or without the addition of NAC significantly dampened these levels. However, the decrease was more prevalent with NAC treatments, but all of the significant benefits were due to the fluids as opposed to NAC. NAC may still further improve microvascular oxygenation by decreasing endothelial glycocalyx damage in addition to fluid resuscitation. In contrast, a clinical study monitored volume loading with HES during elective surgery (20 ml/kg) and observed increased serum glycocalyx biomarkers with HES alone [45]. In our study, fluid resuscitation with HES seemed to be beneficial with regard to these biomarkers levels. One of the main pathways of glycocalyx disruption is thought to be the formation of ROS such as peroxynitrite. As NAC is a well-known scavenger of ROS, we measured the tissue levels of malondialdehyde as a marker of lipid peroxidation. We did not observe a significant decrease when using NAC-supplemented fluid compared to fluid alone. This result can be explained because of the high dose of NAC in our study. Some authors have previously suggested that high doses of NAC may increase MDA levels, whereas lower doses may decrease MDA levels [46]. Additionally, a lack of effect of NAC on lipid peroxidation in cases of established endotoxemia has been shown [47].

### Limitations

In the light of the ongoing debate about the deleterious effects of HES on the kidney, a possible limitation of our study is our use of HES as a resuscitation fluid. However, the present study is a mechanistic study wherein we investigated whether we could ameliorate the inflammatory effects of fluid administration in a sepsis model. In our experience, all fluids cause inflammation [48], and it is the fluid volume that determined the extent of injury. It could be argued that we should have chosen a balanced crystalloid solution instead of a colloid-based solution. However, crystalloid solutions have issues as well. For example, Ringer’s lactate causes even more inflammation than HES [49, 50], and we would also have had to administer a larger volume to maintain blood pressure causing more hemodilution. We chose a colloid solution to keep the amount of fluid required to correct blood pressure to a minimum. We could have used albumin, but even albumin can promote renal failure, as has been shown in a recent study in cardiac surgery [51]. In conclusion, arguably, all fluids have deleterious effects on the kidney to a greater or lesser extent. However, in this proof of concept study, we hope to have introduced the idea that controlling the inflammatory component imposed by fluids such as HES can be implemented by co-administration of an anti-inflammatory drug such as NAC. Our results suggest that such an approach could be used for other fluids, but this approach would require testing in subsequent studies.

## Conclusions

In conclusion, the addition of NAC to fluid resuscitation may improve renal oxygenation and attenuate microvascular dysfunction and AKI. Decreases of renal NO levels and hyaluronic acid levels may be involved in this beneficial effect. A therapeutic strategy combining the macrovascular effects of fluids and the microvascular effects of NAC may be critical to preventing sepsis-induced AKI. This study sets forth a new concept for changing the procedure of fluid resuscitation by the addition of antioxidant therapy during the initial phase of resuscitation.

## Abbreviations

AKI: Acute kidney injury
CaO_2_: Arterial oxygen content
Cl_Crea_: Creatinine clearance
CvO_2_: Renal venous oxygen content
CμPO_2_: Microvascular renal cortical PO_2_
DO_2ren_: Renal oxygen delivery
EFNa^+^: Fractional excretion of sodium
ER: Extraction ratio
HES: Hydroxyethyl starch
L-FABP: Liver-type fatty acid-binding protein
LPS: Lipopolysaccharide
MAP: Mean arterial pressure
MDA: Malondialdehyde
MμPO_2_: Microvascular renal medullar PO_2_
NAC: *N*-Acetylcysteine
NGAL: Neutrophil gelatinase-associated lipocalin
NO: Nitric oxide
RA: Ringer’s acetate
RBF: Renal blood flow
TNa^+^: Total amount of sodium reabsorbed
TNF-α: Tumor necrosis factor-α
VO_2ren_: Renal oxygen consumption
